# The number of obstructive colorectal cancers in Japan has increased during the COVID-19 pandemic: A retrospective single-center cohort study

**DOI:** 10.1016/j.amsu.2020.11.087

**Published:** 2020-12-02

**Authors:** Rei Mizuno, Riki Ganeko, Go Takeuchi, Kazuya Mimura, Hideto Nakahara, Kyoichi Hashimoto, Junsuke Hinami, Takumi Shimomatsuya, Yoshihiro Kubota

**Affiliations:** Department of Surgery, Uji-Tokushukai Medical Center, Uji, 611-0042, 145 Makishima-cho Ishibashi, Uji, Kyoto, Japan

**Keywords:** COVID-19, Colorectal cancers, Bowel obstruction, Screening

## Abstract

**Background:**

The global pandemic of COVID-19 has changed cancer treatment environments. In Japan, cancer screenings were halted and the numbers of endoscopies and surgeries were restricted in some hospitals based on the state of emergency declared. Herein, we investigated the impact of the COVID-19 pandemic on the characteristics of colorectal cancer (CRC) patients in facilities that are on the frontline of both COVID-19 and cancer treatments.

**Patients and methods:**

We retrospectively analyzed the cases of all of the CRC patients (n = 123) who underwent surgery at our regional cancer treatment center and tertiary emergency hospital in Japan during a 120-day period ranging from before to after the state of emergency declaration. CRC patients during the corresponding period in the previous year were also examined.

**Results:**

Although the number of CRC patients did not show a significant change related to the pandemic, the incidence of obstructive CRCs significantly increased after the pandemic's start. The numbers of outpatients and colonoscopies both decreased, which could have resulted in the decrease of CRC patients detected by cancer screening during the pandemic. The numbers of symptomatic CRC patients and emergency admissions both increased significantly during the pandemic.

**Conclusion:**

Our findings indicate the possibility that the discovery of CRCs in patients could be delayed due to the halt in screenings caused by the COVID-19 pandemic, resulting in the increase of obstructive CRCs. These results highlight the importance of cancer screening and suggest that the screening system for cancers should be reorganized before future pandemics.

## Highlights

• The proportion of obstructive CRC patients increased during the COVID-19 pandemic.
• During the COVID-19 pandemic, CRC patients were mainly detected by abdominal symptoms but not by cancer screening.
• It is possible that the delays in the detection and surgeries due to halted screenings promoted the CRC progression.
• The screening systems for cancer should be reorganize before a future pandemic of unknown infectious disease.

## 1 Introduction

The novel coronavirus disease 2019 (COVID-19) which appeared first in Wuhan, China, in November 2019 has spread around the world rapidly [1,2]. In Japan, the first case of COVID-19 was reported on January 15, 2020, and the number of cases increased beginning in late March [3]. In Japan's Kyoto Prefecture, the number of COVID-19 patients began to increase from March 2020. The governor of Kyoto Prefecture declared a state of emergency on April 17, 2020 and urged residents to stay home unless necessary, until the state of emergency was lifted on May 21, 2020. Facing this unprecedented global pandemic, surgical societies and gastroenterological endoscopy societies addressed the general principles of surgical or endoscopic treatments in relation to COVID-19 infection [[4], [5], [6], [7]]. The use of appropriate triage was recommended, and clinicians were urged to consider the postponement or cancellation of gastrointestinal endoscopies or alternative therapeutic approaches such as neoadjuvant chemotherapy or chemoradiation instead of surgery, if possible, for the prevention of nosocomial infections that could result in the breakdown of medical care. Based on the state of emergency, the cancer treatment environment (including diagnoses and surgical treatments) has changed dramatically in Japan and other countries.

Colorectal cancer (CRC) is the most common cancer in Japan [8], and CRCs have been detected by cancer screenings and by the investigations of abdominal symptoms such as abdominal pain, abdominal distention, constipation, and melena. When the state of emergency was declared, Japan's Ministry of Health, Labour and Welfare encouraged the suspension of cancer screenings (in principle) in order to prioritize coronavirus-related treatment. Colorectal cancer screening was resumed with appropriate prevention against COVID-19 on June 1, 2020, following the lifting of the state of emergency on May 21, 2020. We thus speculated that the characteristics of CRC patients during the COVID-19 pandemic could be affected.

Our institution is a regional cancer treatment center and a tertiary emergency hospital in a medical region in Kyoto Prefecture, which has a population of approx. 440,000. Our facilities have been on the frontline of both COVID-19 and cancer management during the COVID-19 pandemic, and patients with COVID-19 have thus been transferred to our hospital and treated during the COVID-19 pandemic.

We conducted the present study to determine the impact of the COVID-19 pandemic on the characteristics of CRC patients and cancer management in a hospital on the frontline of both COVID-19 and cancer treatment, and to report the lessons learned from the COVID-19 pandemic.

## 2 Patients and Methods

### 2.1 Study design and participants

This retrospective single-center cohort study has been registered in a publicly accessible database: Clinical Trials (UMIN000042211). This research was approved by the institutional review board (Code: 2020-16) and has been reported in line with the STROCSS criteria [9].

All the CRC patients who underwent surgery at our hospital during a 120-day period ranging from before to after the declaration of the state of emergency were included in this study. The CRC patients who underwent surgery during the corresponding period in the previous year were also examined. We divided the observation period into four periods as follows. Period 1: December 18, 2018 to April 16, 2019, Period 2: April 17 to August 14, 2019, Period 3: December 19, 2019 to April 16, 2020, and Period 4: April 17 to August 14, 2020. CRC patients who did not undergo surgical resection or patients out of above periods were excluded. All of the CRC patients underwent preoperative chest computed tomography (CT) and/or a polymerase chain reaction (PCR) test to rule out COVID-19 infection. No restriction for surgery was applied during the COVID-19 pandemic with appropriate prevention.

The postoperative follow-up of CRC patients were conducted at our hospital according to the Japanese Society for Cancer of the Colon and Rectum (JSCCR) guidelines 2019 for the treatment of colorectal cancer [10] unless patients wished to postpone the outpatient visit: CRC patients with pStage I –Ⅲ after curative resection were surveyed every 3 months and closer surveillance was conducted in patients allocated curability B due to R1 resection.

### 2.2 Data collection

The collected characteristics of the patients included epidemiological and clinical records, laboratory findings, and pathological findings of CRC patients obtained from the patients' electronic medical records. The numbers of outpatients and patients who underwent diagnostic or screening colonoscopies at our hospital during the observation periods were also collected. The bowel patency was classified as follows. No obstruction: A total colonoscopy was performed. Partial obstruction: A colonoscope could not be passed through the cancer region without symptoms of bowel obstruction that did not require a decompression procedure. Complete obstruction: No passage of a colonoscope, with cancer requiring a decompression procedure with a self-expandable metallic stent or emergency stomas.

### 2.3 Statistical analyses

Poisson's test was used to compare the emergency admission rate and the incidence of obstructive CRCs between periods 1–3 and period 4. The Kruskal-Wallis test was used for the comparison of the distance between the residences of patients and our hospital. All statistical analyses were performed using R ver. 3.6.3 (http://r-project.org/). P < 0.05 was considered to be a significant difference.

## 3 Results

### 3.1 Patient characteristics

**Fig. 1:**
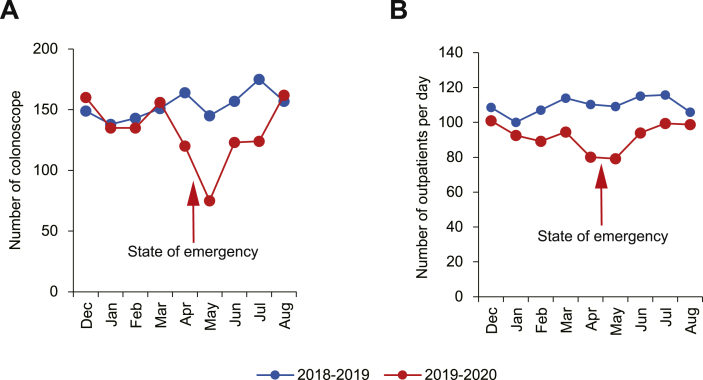
Changes in the numbers of screening or diagnostic colonoscopies and outpatients in our hospital. A: Total numbers of screening or diagnostic colonoscopies in each month. **B:** The average numbers of daily outpatients in each month. *Blue lines:* 2018–2019 data. *Red lines:* 2019–2020 data.

The study population was 123 CRC patients who underwent surgeries during the observational periods. Although our hospital has been accepting patients with COVID-19, none of these CRC patients tested positive for COVID-19. Based on the prefecture's state of emergency, healthcare facilities were encouraged to suspend their cancer screenings and screening or diagnostic endoscopies. In fact, the number of outpatients who came to our department and the number of screening or diagnostic colonoscopies performed at our hospital both significantly decreased after the declaration of the state of emergency (Fig. 1). We thus suspected that the number of CRC patients undergoing colorectal resection would be decreased during the COVID-19 pandemic.

Contrary to our expectations, no significant change was observed in the number of CRC patients who underwent surgeries at our hospital. However, the number of patients who needed an emergency admission and that of patients with obstructive CRCs were significantly increased in period 4. The incidence of complete obstruction at our hospital was roughly 15% before the state of emergency, whereas it increased significantly to 39% during the COVID-19 pandemic (p < 0.05). The incidence of partial and complete obstruction was significantly increased to 67% in period 4, whereas it was 19%–42% in the previous three periods. Consequently, the number of patients with CRC at an advanced pathological stage tended to increase in period 4 (Table 1).

**Table 1:** The characteristics of the patients who underwent surgery for CRC during a 120-day period related to the COVID-19 pandemic. C: cecum, A: ascending colon, T: transverse colon, D: descending colon, S: sigmoid colon, R: rectum. The Poisson's test was used to compare the emergency admission rate and the incidence of obstructive CRCs between periods 1–3 and period 4. *p < 0.05.

		Period 1 Dec 18, 2018 to Apr 16, 2019 (n = 33)	Period 2 Apr 17–Aug 14 2019 (n = 29)	Period 3 Dec 19, 2019 to Apr 16, 2020 (n = 30)	Period 4 Apr 17–Aug 14, 2020 (n = 31)
Age, yrs; mean ± SD		70.5 ± 10.6	75.9 ± 11.6	72.7 ± 9.1	72 ± 10.7
Female		11 (33.3%)	12 (41.4%)	15 (50.0%)	16 (51.6%)
Emergency admission		6 (18.2%)	4 (13.8%)	4 (13.3%)	12 (38.7%) *
Hb, g/dL; mean ± SD		11.8 ± 2.9	12.3 ± 2.1	11.8 ± 2.3	12.5 ± 2.8
CEA, ng/ml; mean ± SD		38.6 ± 118.5	20.5 ± 48.1	48.3 ± 208.9	28.5 ± 59.1
Tumor location C/A/T/D/S/R		4/5/6/0/12/9	1/7/2/1/10/10	3/6/2/2/9/12	6/5/2/1/11/7
Bowel patency	No obstruction	19 (57.6%)	23 (79.3%)	20 (66.7%)	10 (32.3%)
	Partial obstruction	9 (27.3%)	2 (6.9%)	5 (16.7%)	9 (29.0%)
	Complete obstruction	5 (15.2%)	4 (13.8%)	5 (16.7%)	12 (38.7%) *
PStage	Stage 0	3	2	0	1
	Stage I	3	8	5	2
	Stage II (a/b/c)	9/6/2	8/1/0	6/1/2	5/4/1
	Stage III (a/b/c)	2/4/2	1/7/1	0/6/7	1/4/7
	Stage IV (a/b/c)	1/1/2	3/0/0	4/0/0	2/1/3

### 3.2 The delays in the detection of CRCs

**Fig. 2:**
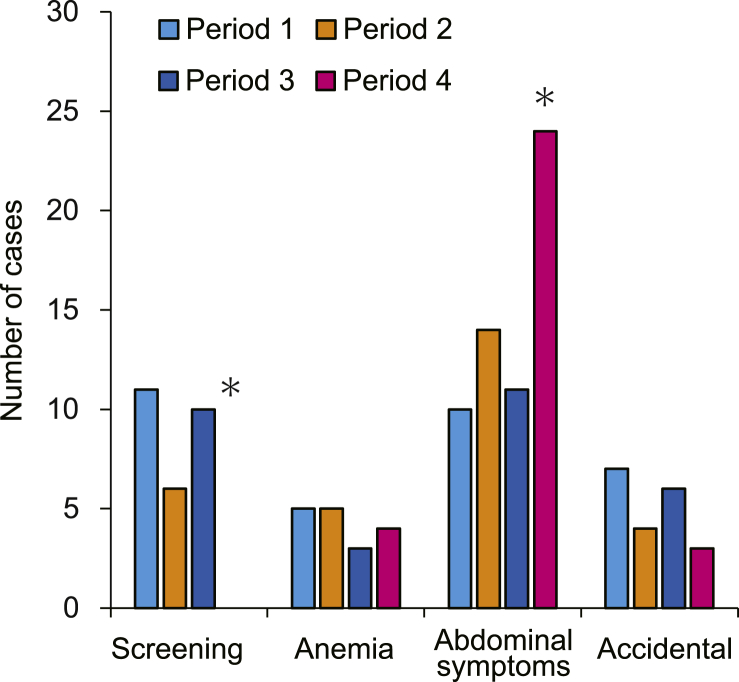
Events that contributed to the detection of CRCs. Poisson's test was used to compare the data in periods 1–3 and those in period 4. *p < 0.05.

We next investigated the reasons why the number of obstructive CRCs increased during the COVID-19 pandemic, and two possibilities arose. One possibility is that halting the screening could have delayed the detection of CRCs. As shown in Fig. 1, the numbers of outpatients and screening or diagnostic colonoscopies performed at our hospital started to decrease as COVID-19 started to spread in Kyoto Prefecture in March 2020. This tendency continued for a while, and the numbers began to increase again when the state of emergency was lifted on May 21, 2020 (Fig. 1). We also examined the events contributing to the detection of CRCs, and we observed that in period 4 the number of patients detected by screening dropped to zero, and the number of patients with abdominal symptoms increased significantly (Fig. 2).

Taken together, our results may indicate that the delays in the detection of and surgery for CRC due to the suspended cancer screenings may have promoted the progression of CRCs, resulting in the increases in the numbers of symptomatic patients and obstructive CRCs.

### 3.3 Patients' selection of which hospital to consult

**Fig. 3:**
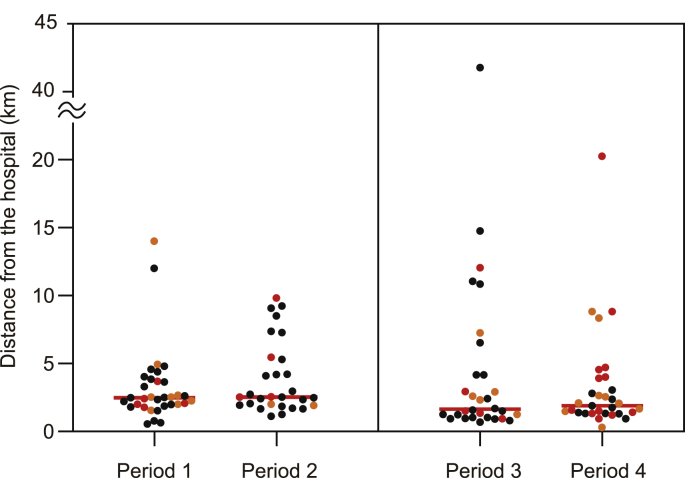
Distances between the residences of the patients who underwent surgery at our hospital during the designated periods. Each dot indicates the distance between a patient's residence and our hospital. Red dots: Patients with complete bowel obstruction, orange: partial obstruction, and black: no obstruction. Red bars: The mean value. The Kruskal-Wallis test was used for the statistical analysis. No significant change in patient residences during the study period was observed. (For interpretation of the references to color in this figure legend, the reader is referred to the Web version of this article.)

Another possible reason for the increase of obstructive CRCs is that COVID-19 could have changed the patients' selection of which hospital to consult about their condition. Some hospitals in Japan limited or halted surgeries (including emergency surgeries) to prevent nosocomial infection; we did not restrict surgeries because our hospital is a regional cancer treatment center and a tertiary emergency hospital with adequate medical staff and appropriate prevention against COVID-19. Therefore, patients who would have otherwise been treated at nearby hospitals might have been more likely to come to our hospital. To investigate this possibility, we examined the distances between the patients' residences and our hospital and found that the patients' locations did not show any significant changes from the pre-pandemic scenario (Fig. 3). This result indicates that the change in patients' location within their medical service area did not strongly affect the increase in the number of obstructive CRCs during the COVID-19 pandemic.

## 4 Discussion

The global COVID-19 pandemic has changed the environment for cancer treatment. Surgical societies and gastroenterological endoscopy societies have recommended appropriate triages for the indication of screening, endoscopy, and surgery and have encouraged alternative therapeutic approaches [[4], [5], [6], [7]]. Alboraie et al. performed an international survey to investigate the global impact of COVID-19 on gastrointestinal endoscopy units, and they reported that the number of gastrointestinal endoscopic procedures performed during the COVID-19 pandemic was significantly decreased [11]. Studies from several countries have reported a reduction in surgical admissions during the COVID-19 pandemic [[12], [13], [14], [15], [16], [17]]. Nunoo-Mensah et al. conducted a survey concerning the global effects of COVID-19 on colorectal practice and surgery, and they reported that COVID-19 has affected the ability of colorectal surgeons to offer care to their patients. They demonstrated that 61% of the surgeons were prepared to defer the elective surgeries for CRCs, with 29% willing to defer for ≤8 weeks [18].

In the present study, the number of outpatients and the number of screening or diagnostic colonoscopies performed at our hospital both fell after the declaration of the state of emergency. We thus expected that the number of CRC patients undergoing a colorectal resection at our hospital would be decreased during the COVID-19 pandemic. However, our analyses revealed no significant change in the number of CRC patients. This could be because our hospital is a designated cancer hospital and a tertiary emergency hospital in its medical service area, and no restriction of surgery was applied with appropriate prevention and preoperative testing with chest CT and/or PCR for COVID-19.

Although the total number of CRC patients did not show any significant change in this analysis, the number of patients who needed an emergency admission and the number of patients with obstructive CRCs both increased significantly during the COVID-19 pandemic. Almost all of the emergency admissions for CRC patients during the observation periods were due to a bowel obstruction which required decompression procedures. The reported incidence of obstructive CRCs requiring decompression procedures is 10%–20% [19,20], which is compatible with the incidence of CRCs with complete obstruction before the state of emergency in our present investigation. However, we observed that the incidence of obstructive CRCs requiring decompression significantly increased to 39% during the COVID-19 pandemic. Moreover, after the state of emergency was declared, the number of CRC patients detected by the screening dropped to zero and the number of symptomatic CRC patients increased significantly. McLean et al. conducted a single-center observational cohort study in United Kingdom and reported that the incidence of gastrointestinal obstruction was significantly increased after the lockdown imposed by the UK government, although they did not clarify the reasons for the bowel obstructions [12].

A possible reason for the increase in the number of obstructive CRCs in this study could be that the suspension of cancer screenings encouraged by Japan's Ministry of Health, Labour and Welfare under the principle of prioritizing coronavirus-related treatment resulted in the progression of CRCs. The stay-home request by the governor of Kyoto Prefecture could also have affected the delay in the diagnosis of CRCs. In fact, the number of colonoscopies performed at our hospital and the number of outpatients were decreased during the COVID-19 pandemic, and they increased after the lifting of the state of emergency. Turaga et al. reported that a postponement of surgery in CRC patients by > 4 weeks influenced the patients' prognoses [21]. Although we did not conduct a detailed survey regarding the patients' selection of hospitals, our analysis of the patients' locations in the medical service area did not seem to show any significant change during the COVID-19 pandemic. Taken together, our findings indicate that rather than a change in patients' location in the medical service area, it was the delayed detection that could have contributed to the progression and the increase of obstructive CRCs.

Our study is limited as an observational study conducted at a single center. A region-wide or nationwide multi-center study should be conducted to determine whether the COVID-19 pandemic has contributed to the progression of CRCs and whether the decrease in the number of screenings has truly contributed to the progression of diseases, including CRC. The relocations of CRC patients in each medical region should be examined as part of attempts to elucidate the precise reason(s) for the progression of CRCs during the COVID-19 pandemic.

## 5 Conclusions

Our findings indicate the possibility that the discovery of CRCs in patients who might be saved could be delayed due to the halt in screenings caused by the COVID-19 pandemic, which highlights the importance of cancer screening. A reorganization of the screening system for cancer before future pandemics of unknown infectious disease seems advisable, taking the lessons learned from the COVID-19 pandemic.

## Declaration of competing interest

There are no conflicts of interests.

## Registration of research studies

1. Name of the registry: The impact of COVID-19 pandemic on colorectal cancer therapy.
2. Unique Identifying number or registration ID: UMIN000042211
3. Hyperlink to your specific registration (must be publicly accessible and will be checked): https://upload.umin.ac.jp/cgi-open-bin/ctr/ctr_view.cgi?recptno=R000048176
